# Inclusion of cow records in genomic evaluations and impact on bias due to preferential treatment

**DOI:** 10.1186/1297-9686-44-40

**Published:** 2012-12-27

**Authors:** Romain Dassonneville, Aurelia Baur, Sébastien Fritz, Didier Boichard, Vincent Ducrocq

**Affiliations:** 1 INRA, UMR1313 Génétique Animale et Biologie Intégrative, Jouy-en-Josas F-78350, France; 2 Institut de l’Elevage, 149 rue de Bercy, Paris F-75595, France; 3 UNCEIA, 149 rue de BercyParis F-75595, France

## Abstract

**Background:**

Today, genomic evaluations are an essential feature of dairy cattle breeding. Initially, genomic evaluation targeted young bulls but recently, a rapidly increasing number of females (both heifers and cows) are being genotyped. A rising issue is whether and how own performance of genotyped cows should be included in genomic evaluations. The purpose of this study was to assess the impact of including yield deviations, i.e. own performance of cows, in genomic evaluations.

**Methods:**

Two different genomic evaluations were performed: one including only reliable daughter yield deviations of proven bulls based on their non-genotyped daughters, and one including both daughter yield deviations for males and own yield deviations for genotyped females. Milk yield, the trait most prone to preferential treatment, and somatic cell count, for which such a bias is very unlikely, were studied. Data consisted of two groups of animals from the three main dairy breeds in France: 11 884 elite females genotyped by breeding companies and 7032 cows genotyped for a research project (and considered as randomly selected from the commercial population).

**Results:**

For several measures that could be related to preferential treatment bias, the elite group presented a different pattern of estimated breeding values for milk yield compared to the other combinations of trait and group: for instance, for milk yield, the average difference between estimated breeding values with or without own yield deviations was significantly different from 0 for this group. Correlations between estimated breeding values with or without yield deviations were lower for elite females than for randomly selected cows for milk yield but were very similar for somatic cell count.

**Conclusions:**

This study demonstrated that including own milk performance of elite females leads to biased (over-estimated) genomic evaluations. Thus, milk production records of elite cows require specific treatment in genomic evaluation.

## Background

Preferential treatment and the bias it induces is a long-standing issue in genetic evaluation. Preferential treatment can be defined as management practices that modify production. Such practices are selective since they are applied to some cows but not to most of their herd mates [1] and may be related to housing, feeding or reproduction systems. The bias induced by preferential treatment was initially detected by inconsistencies between the parent average breeding value of a bull (influenced by the breeding value of its dam) and performance of the bull’s daughters (included to estimate the bull’s breeding value after progeny testing known to be less influenced by its dam) [2].

With the development of genomic evaluation, the issue of bias due to preferential treatment is highly relevant again. In genomic evaluation, the reference population consists of individuals with both genotypes and performance records and which are used to estimate marker effects. The larger the reference population size, the more reliable the genomic evaluations [3]. In the early years of genomic evaluation, reference populations consisted of progeny-tested bulls only and genomic evaluations were based only on reliable averaged performances of each bull’s daughters. Considering the rapidly increasing number of genotyped cows with own records, it is very appealing to include these genotyped cows in the reference population. Moreover, this will become necessary in the future, to upgrade the reference population if the number of bulls with a progeny evaluation declines. Within the female population, potential bull dams have been the first target for genotyping. The use of potentially biased records of these genotyped elite females in genomic evaluation may have two major impacts, i.e. (1) on the genomically enhanced breeding values (GEBV) of these cows and their relatives and (2) on the prediction equations.

Strategies to deal with this issue differ between countries. For example, the USA chose very early to include genotyped cows in the reference population [4], whereas, fearing potential bias, Canada and the Eurogenomics consortium [5] decided not to include cows in the reference population. Later, Wiggans et al. [4] proposed a method to adjust records on genotyped females prior to their use in genomic evaluation, which has since been applied in the USA. There is a need to more precisely assess the impact of including records on genotyped cows in the reference population on the reliability of genomic predictions.

With a reference population consisting of AI (artificial insemination) bulls, the phenotypic information of a bull’s daughters is summarized by the daughter yield deviation (DYD) as defined by VanRaden and Wiggans [6]. DYD represent the average performance of a bull’s daughters corrected for all fixed effects (such as herd, year, season among others), the permanent environment effect, and also for the genetic contribution of the bull’s mate (i.e., half the additive genetic value of the cow’s dam). Subsequently, the DYD are used as if they are the performance of the bulls themselves.

The equivalent phenotypic measure for females is the yield deviation, YD, which corresponds to the performance of the cow itself (not its progeny’s), also corrected for all effects except the genetic effect.

In a preliminary study in which the own record of genotyped females were either included or excluded (results not shown), the correlations between phenotypic (DYD) values and GEBV for bulls of the validation population were evaluated for several production traits and for somatic cell count (SCC). This value can be regarded as the square root of the realized reliability. These correlations did not decrease when the own performance records of the genotyped cows, which are ancestors of the validation bulls, were removed from the training set, suggesting two opposite interpretations. On one hand, removing female information did not result in a loss in accuracy, which means that this additional information was (at least partly) biased, providing as much noise as useful information. However, on the other hand, one would expect an increase in the correlation if the removed information had been heavily biased.

The objective of this study was to compare predictions obtained with two different genomic evaluations. In the first, only bulls were included in the reference population, while in the second, genotyped cows were added to the reference population. Two traits (milk yield and SCC) were considered that differ in the preferential treatment applied. In contrast with other studies, including our preliminary work, two distinct cow populations were considered, one including only elite dams and one including genotyped cows that were (nearly) “randomly selected” from the commercial population. The latter group is expected to be less affected by preferential treatment or by selective genotyping. Under the assumption that a bias is induced by preferential treatment, GEBV are expected to have different characteristics for the elite cow population and milk yield compared to the other combinations of trait and population.

## Methods

### Data

This study focused on genotyped dairy cows. Three French dairy breeds were investigated separately in this study: Holstein, Montbéliarde, and Normande. The impact of two cow populations was investigated: elite females and “randomly selected” cows. Females (both heifers and cows) genotyped by breeding companies were considered as elite females. It was assumed that if a breeding organization was interested in genotyping a particular female as a potential bull dam, and was ready to pay for its GEBV, this female could be defined as “elite”. As a reference, a non-preferentially treated group, i.e. a representative subset of the commercial population was needed. For a research project on genetic and environmental parameters of milk fatty acids composition, 7032 dairy cows that were specifically chosen to be representative of the commercial population were genotyped. The choice of the genotyped cows was based on the following constraints: from a set of sires (20 for each of the Normande and Montbéliarde breeds and 30 for the Holstein breed) a limited number of daughters per sire were randomly selected within a given set of participating herds. We considered these cows as “randomly selected”. Elite cows are more likely to be preferentially treated and have records that are biased upward than the “randomly selected” group of cows that are less prone to such a bias. Because elite cows were genotyped before their first calving, i.e., before any own performance was known, potential selective genotyping was limited to pedigree information on total merit index, which includes both production and SCC.

Obviously, the reference populations also included progeny-tested bulls, distributed across several generations. Table 1 summarizes the number of genotyped bulls, elite females and “randomly selected” cows in the three breeds. In total, 1798, 2157 and 19 485 genotyped progeny-tested bulls were included in the reference population for the Normande, Montbéliarde and Holstein breeds, respectively. The Normande and Montbéliarde reference bull populations only included individuals genotyped in France whereas the Holstein reference bull population also included bulls genotyped by European partner breeding organizations that exchange genotype information within the Eurogenomics consortium [5]. All females and most males were genotyped with the Bovine 50K chip (Illumina inc., San Diego), while some males from the Dutch population were genotyped with the CRV (The Netherlands) 60K custom chip and imputed.

**Table 1:** Number of genotyped individuals for the three breeds for three groups of individuals

**Breed**	**AI bulls**	**Elite cows**	**Randomly selected cows**
Montbéliarde	2157	2190	1826
Normande	1798	2129	2374
Holstein	19 485	7565	2832

AI = artificial insemination.

### Performances included for the reference population

For each breed, two kinds of genomic evaluations were computed. In the first, only males were included in the training population and only DYD were used to estimate marker effects. In the second, genotyped cows with own records were added to the training population and phenotypic data on both males and some females were used to estimate marker effects.

Both YD and DYD are by-products of the official polygenic evaluations. Both milk yield and SCC were evaluated with an animal model (polygenic, pedigree-based) evaluation. For milk yield, heterogeneity of variances was accounted for, as described by Robert et al. [7]. YD and DYD were then corrected for heterogeneity of variance and expressed as in a standardized (reference) environment. Data used for this study were obtained after the official evaluation of November 2011 and were used as inputs for the genomic evaluations. When YD were used for genotyped cows, their contribution was removed from their sires’ DYD in order to avoid double counting of their records.

### Genomic evaluation model

The French genomic evaluation model is an extension of the marker-assisted evaluation approach of Fernando and Grossman [8], using haplotypes of three to five single nucleotide polymorphisms (SNP). The QTL-BLUP model can be written as:

$$y_{i}=\mu +u_{i}+\sum_{j=1}^{\mathrm{nQTL}}\left(h_{\mathrm{ij}}^{p}+h_{\mathrm{ij}}^{m}\right)+\;e_{i}$$

where μ is a constant, y_i_ is the phenotypic observation (2xDYD or YD) of individual i, u_i_ is its random (pedigree-based) residual polygenic effect, h_ij_^p^ and h_ij_^m^ are the random effects of the paternal and maternal haplotypes for QTL j, and e_i_ is the residual, with heterogeneous residual variances. Since the model is an individual animal model, it was assumed that phenotypes were obtained for each animal and their weights were expressed in equivalent number of records.

The QTL included in the model were selected by a combination of two approaches [9]. First, several dozens of QTL per trait were detected after QTL fine-mapping using a linkage disequilibrium linkage analysis (LDLA), as described by Druet et al. [10]. Then, hundreds of haplotypes were chosen using the Elastic Net algorithm [11]. Finally, between 327 and 726 QTL for milk yield and between 404 and 525 QTL for SCC were included in the model depending on the breed.

To estimate the GEBV, all individuals were included together. Elite and “randomly selected” subsets were analyzed separately, but the GEBV of individuals present in these two populations came from the same evaluation.

## Results

Mean and standard deviations of national evaluation EBV for the two groups of cows (elite and “randomly selected”) are reported in Table 2. This table underlines the differences in genetic merit between the two groups for the traits of interest, i.e. the average GEBV ranged from 359 to 737 kg for milk yield (corresponding to 0.5 to 0.95 genetic standard deviations) and from 0.22 to 0.45 genetic standard deviations for SCC. For both traits, the elite population presented a genetic superiority over the “randomly selected” population. The difference was larger for milk yield than for SCC. As already pointed out, some selective genotyping (based on parent average only) may exist in the elite population for both traits.

**Table 2:** Statistics of official national estimated breeding values for the elite and “randomly selected” groups of cows

		**Elite**		**Random**	
	**Breed**	**Mean**	**SD**	**Mean**	**SD**
Milk yield (kg)	Montbéliarde	663	348	304	320
	Normande	717	318	203	333
	Holstein	1055	462	318	386
SCC	Montbéliarde	0.26	0.69	0.04	0.59
	Normande	0.29	0.64	-0.16	0.73
	Holstein	0.40	0.63	-0.05	0.69

SD = standard deviation; SCC = somatic cell count expressed in genetic standard deviation; number of cows are given in Table 1.

Correlations between GEBV obtained by including either both bulls’ DYD and cows’ YD (GEBV_(DYD+YD)_) in the reference population or only DYD (GEBV_(DYD)_) are shown in Table 3. Under the assumption that a bias was induced by preferential treatment only in the case of milk production for elite cows, these correlations should be similar between the elite and “randomly selected” groups for SCC, but for milk yield, they should be lower for the elite group than for the “randomly selected” group. For the Normande breed and SCC, the correlations were essentially the same in the elite and “randomly selected” cows (difference below 0.01). However, for milk yield, the correlation was substantially higher for the “randomly selected” cows than for the elite group (0.82 instead of 0.77). For the Montbéliarde breed, the correlation was also higher for the “randomly selected” group for milk yield (0.77 instead of 0.74), but for SCC it was lower for the “randomly selected” group than for the elite group (difference of 0.02). For the Holstein breed, differences in correlations between the two groups were almost null (0.001) for SCC and very small (0.007) for milk yield.

**Table 3:** Correlations between GEBV obtained when including both DYD and YD and when including only DYD for the elite and “randomly selected” groups of cows

**Breed**	**Trait**	**Elite cows**	**Randomly selected cows**
Montbéliarde	Milk yield	0.740	0.770
	SCC	0.915	0.893
Normande	Milk yield	0.768	0.820
	SCC	0.900	0.909
Holstein	Milk yield	0.931	0.938
	SCC	0.966	0.965

GEBV = genomically estimated breeding values; DYD = daughter yield deviations; YD = yield deviations; SCC = somatic cell count.

**Figure 1:**
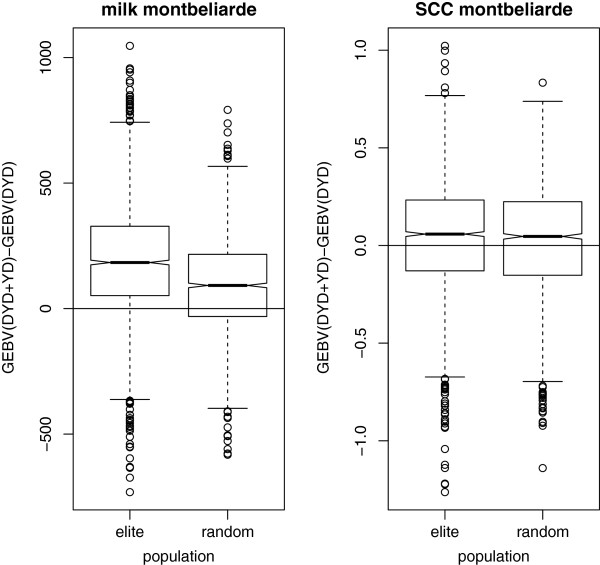
**Box plot of differences between GEBV including both DYD and YD and GEBV including DYD only for the elite and “randomly selected” Montbéliarde cow groups.** Milk yield in kg; SCC = somatic cell count expressed in genetic standard deviation.

**Figure 2:**
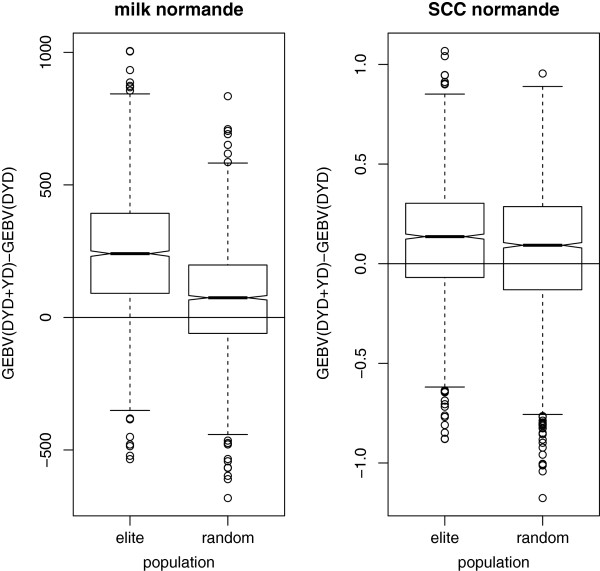
**Box plot of differences between GEBV including both DYD and YD and GEBV including DYD only for the elite and “randomly selected” Normande cow groups.** Milk yield in kg; SCC = somatic cell count expressed in genetic standard deviation.

**Figure 3:**
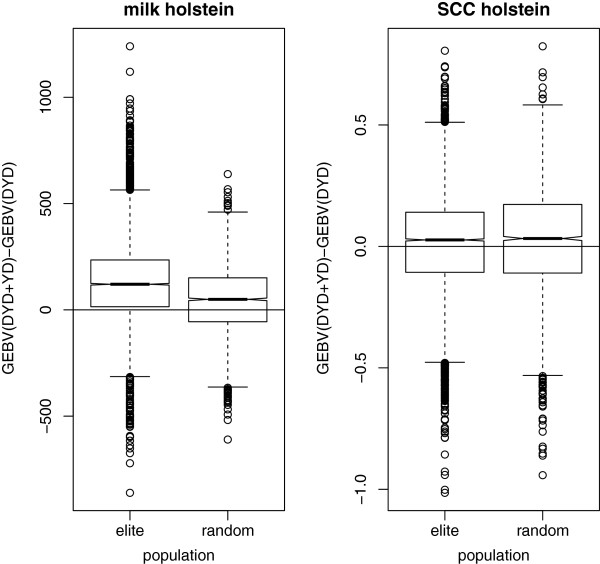
**Box plot differences between GEBV including both DYD and YD and GEBV including DYD only for the elite and “randomly selected” Holstein cow groups.** Milk yield in kg; SCC = somatic cell count expressed in genetic standard deviation.

Box plots of the differences between GEBV_(DYD+YD)_ and GEBV_(DYD)_ for the three breeds are presented in Figures 1, 2, 3. For each breed, four box plots are displayed, for each combination of trait and population. Note that the values are expressed in kg for milk yield (one genetic standard deviation equals 591, 661 and 759 kg for Normande, Montbéliarde and Holstein breeds, respectively) and in genetic standard deviations for SCC. In the absence of bias in the records on cows, these box plots should be centered around 0 and symmetrically distributed.

For each breed, the same pattern was observed: three box plots out of four had a mean close to 0. The null value was included in the second quartile for SCC for both groups of cows but only for the “randomly selected” group for milk yield. For milk yield, GEBV for the elite cows clearly presented a different pattern; the box was entirely above 0, meaning that GEBV_(DYD+YD)_ was greater than GEBV_(DYD)_ for more than 75% of the cows. This was not observed for SCC. Note that the median is not strictly equal to 0 for any of the four box plots, although it was very close to 0 for SCC in the Holstein breed. The elite cows also presented a higher variability of differences for milk yield, especially in the Montbéliarde and Holstein breeds.

Table 4 focuses on the average differences between GEBV_(DYD+YD)_ and GEBV_(DYD)_. Under the assumption that a bias was induced by preferential treatment only in the case of milk yield for elite cows, this difference should be 0 for the “randomly selected” group or for SCC but significantly larger than 0 for the elite group for milk yield. Across breeds, these average differences between GEBV_(DYD+YD)_ and GEBV_(DYD)_ were close to 0 and similar when comparing “randomly selected” and elite groups for SCC. They were equal to 0.01 and 0.02 genetic standard deviations for the Holstein and Montbéliarde breeds, respectively. For the Normande breed and SCC, the elite group had a slightly higher average difference than the “randomly selected” group (0.11 instead of 0.06). Indeed, these values were again not strictly equal to 0. However, for milk yield, this average difference was much larger for the elite group than for the “randomly selected” group, i.e. about 2, 3 and 4 times greater for the Montbéliarde, Holstein, and Normande breeds, respectively. In absolute terms, for the Normande breed, the average difference was 0.3 genetic standard deviations greater for the elite group than the “randomly selected” group.

**Table 4:** Average difference (in genetic standard deviation) between GEBV calculated with and without YD for the elite and “randomly selected” cow groups

**Breed**	**Trait**	**Elite cows**	**Randomly selected cows**
Montbéliarde	Milk yield	0.286	0.135
	SCC	0.040	0.020
Normande	Milk yield	0.409	0.117
	SCC	0.110	0.060
Holstein	Milk yield	0.168	0.060
	SCC	0.013	0.024

SCC = somatic cell count.

## Discussion

Key assumptions in our study were that heifers and cows genotyped by breeding companies are elite females and that the cows genotyped for the research project could be considered as representative of the commercial population. Indeed, each breeding company has its own strategy for bull dam selection: some companies genotype a relatively large proportion of the population and select on a broad basis, while others are more selective and only genotype top females based on their total merit index. Different breeding companies may put a different emphasis on different traits, or the sire analysts may focus on a limited number of maternal cow families, which are more likely to be affected by preferential treatment. For the cows genotyped in the research project, even if the sires (constraint set on number of genotyped progeny) were the most used within each breed, this group of cows may not be a perfect random sample of the commercial population. However, the two groups of cows are easily identified when considering the average EBV of the cows, since the elite group presented a superiority of 0.4 to 1 genetic standard deviations for each breed and trait. The lowest difference in EBV between the elite and “randomly selected” groups was observed in the Montbéliarde breed, likely because the objective of the main breeding organization involved in this breed is to genotype a large proportion of candidates from the whole population. Thus, the elite group includes some females that would not strictly fit with a selection criterion mainly based on milk yield EBV for the Montbéliarde breed. It should also be emphasized that elite cows were genotyped before their first calving, thus, it is unlikely that preferential treatment occurred for all elite cows, especially those with a disappointing GEBV.

Milk yield is obviously a more important trait in the breeding goal, which explains why the superiority of the elite group over the “randomly selected” group is greater for this trait. However, the elite group was also genetically superior for SCC. Thus any difference in results between the two traits when comparing the two female groups cannot be explained only by the genetic superiority of the elite group.

The two traits not only differ in nature (a production trait *vs* a health trait) but also in their heritability, which is 0.3 for milk yield and 0.15 for SCC in our case. This difference in heritability has an impact on the amount of information that a cow’s own performance contributes to its GEBV; own performance will have a larger impact on the cow’s GEBV for milk yield because of this higher heritability.

A main feature of the genomic prediction model is that polygenic effects (based on pedigree) and haplotype effects (based on marker information) are estimated jointly. This is useful to properly estimate both terms, compared with blending procedures [12] for instance. However, both polygenic and haplotype effects may be affected by biases in the phenotypic data used.

Correlations between GEBV_(DYD+YD)_ and GEBV_(DYD)_ presented a different pattern between milk yield and SCC. Indeed, except for the Montbéliarde breed, these correlations were very similar for the elite and “randomly selected” groups for SCC but for milk yield, the decrease in correlation was lower for the elite group than for the “randomly selected” group (difference of up to 0.04). This provides the first evidence of the existence of a bias induced by including own phenotypes of genotyped cows in genomic evaluation.

Correlations were also higher for SCC than for milk yield. However, as already mentioned, this can be mainly explained by the lower heritability of SCC. Indeed, the information that is added by a cow’s own phenotype (YD) is less for a lowly heritable trait and is, therefore, expected to result in smaller changes in GEBV.

The reference population for the Holstein breed was much larger than that for the two other breeds. This results in more precisely estimated marker effects and more stable genomic evaluations for the Holstein breed. This may be the reason why correlations of GEBV were higher for this breed, regardless of the group and trait considered. This may also explain why the differences observed between the elite and “randomly selected” groups were smaller for this breed.

Differences between GEBV_(DYD+YD)_ and GEBV_(DYD)_ were computed for the two traits for each cow in the two groups. Graphical representations of these differences (Figures 1, 2, 3) clearly showed a different pattern for milk yield for the elite group compared to the “randomly selected” group and for SCC. A large fraction of the elite cows presented a positive difference for milk yield, meaning that including their own record in genomic predictions led to an increase of their GEBV. This phenomenon was not observed for SSC or for the “randomly selected” group, for which differences were almost equally distributed between positive and negative values.

When expressed in genetic standard deviation units, the average differences observed confirmed that the elite group for milk yield presented different characteristics than the “randomly selected” group or for SCC. Admittedly, the mean values were not strictly equal to 0 for the “randomly selected” group or for SCC, and it is difficult to explain why. However, the mean difference was up to 0.3 genetic standard deviations (in the Normande breed) higher for the elite group than for the “randomly selected” group.

The elite group was also genetically superior for SCC but no real difference between GEBV_(DYD+YD)_ and GEBV_(DYD)_ was observed in either the elite or the “randomly selected” groups. This means that the systematic overestimation of GEBV observed when milk yield YD are included is induced by inflated performances of the elite group. This clearly demonstrates the existence of a bias of GEBV for milk yield but not for SCC. Preferential treatment is the most immediate explanation for this bias, although we cannot exclude some genetic underestimation of the elite cows in our models which did not account for the records of the dams.

Our findings were obtained considering a group of cows as a whole. This does not mean that every single individual of this group has inflated performances. In particular, the GEBV decreased for a significant proportion of the elite cows when their own YD were included.

Wiggans et al. [4] also demonstrated the existence of a bias in genomic evaluations when using unadjusted records for genotyped cows in the reference population. Indeed, for milk yield in Holsteins, the regression coefficient of the progeny test EBV of bulls on their GEBV prior to progeny testing decreased when unadjusted records for genotyped cows were included. The regression coefficient is a measure of how inflated GEBV are compared to EBV and showed a bias equal to 50 kg. The realized reliability, calculated as the squared correlation between GEBV and deregressed proofs for bulls of the validation population, was also lower when records on cows were included. Furthermore, they also observed a bias in genomic predictions equations, as marker effects of the X chromosome presented a specific pattern, suggesting that females behaved systematically differently than males.

Since a bias from including records on genotyped cows has now been demonstrated in genomic evaluations, it is necessary to develop methods to correct it. The first solution is to not use own records of genotyped cows. It is possible to estimate direct genomic breeding values obtained using a reference population consisting of bulls only, or to use GEBV (obtained after blending for instance) in which the polygenic component only includes performance (DYD) of male relatives. However, such a solution is not completely satisfactory. First, the AI industry may want to include own records of genotyped cows even if it does not increase reliabilities of genomic evaluations. Secondly, and more importantly, this solution implies that a large amount of potentially valuable information is not used. With the release of an efficient low density SNP chip [13] to genotype females at a reduced cost, one can expect that many heifers from commercial herds will be genotyped in the near future, providing a large number of genotyped cows. Obviously, for most of these commercial animals, records are likely to be unbiased and they will build up the reference population of the future.

Another solution is to adjust (i.e. pre-correct) the own phenotype of genotyped cows before their inclusion in genomic evaluation. This is the option retained by Wiggans et al. [4], who proposed to adjust the mean and variances of the estimated Mendelian sampling term of genotyped cows, such that they are similar to those of bulls. Interesting improvements in several measures related to bias of GEBV and prediction equations were reported. However, whether they are adjusted or not, it is not really possible to distinguish a positive Mendelian sampling from a bias due to preferential treatment for records of cows.

Single-step procedures [14] are appealing because non-genotyped individuals benefit from marker information of their genotyped relatives. It has also some interesting properties in terms of bias due to pre-selection of young bulls. However, solutions to remove bias induced by preferential treatment (such as blending, or adjustment of Mendelian sampling terms) are still needed.

## Conclusions

We compared genomic predictions obtained after genetic evaluations with or without including records of genotyped cows in the reference population. Results showed that when genotyped cows belonged to the group of elite cows, their GEBV for milk yield presented a different pattern than when they represent a random sample of the commercial population, whereas these two groups of cows showed similar characteristics for SCC. Correlations between GEBV computed with or without cows in the reference population were lower for the elite group for milk yield but not for SCC. A systematic overestimation of GEBV was also observed when the own milk yield records of the elite cows were included in the reference population. This study demonstrates that explicitly including own records of elite females results in biased (overestimated) GEBV.

## Competing interests

The authors declare that they have no competing interests.

## Authors’ contributions

RD performed the analysis and wrote the manuscript. RD, VD, SF and DB designed the study. AB computed genomic evaluations. All authors read and approved the final manuscript.
